# Spin-Space-Encoding Magnetic Resonance Imaging: A New Application for Rapid and Sensitive Monitoring of Dynamic Swelling of Confined Hydrogels

**DOI:** 10.3390/molecules28073116

**Published:** 2023-03-30

**Authors:** Rui Wang, Jiaxiang Xin, Zhengxiao Ji, Mengni Zhu, Yihua Yu, Min Xu

**Affiliations:** Shanghai Key Laboratory of Magnetic Resonance, School of Physics and Electronic Science, East China Normal University, Shanghai 200241, China; 51184700048@stu.ecnu.edu.cn (R.W.); 52174701014@stu.ecnu.edu.cn (J.X.); 51214700058@stu.ecnu.edu.cn (Z.J.); 52214700018@stu.ecnu.edu.cn (M.Z.); yhyu@phy.ecnu.edu.cn (Y.Y.)

**Keywords:** gradient broadening, profile, hydrogel, swelling

## Abstract

An NMR method based on the gradient-based broadening fingerprint using line shape enhancement (PROFILE) is put forward to precisely and sensitively study hydrogel swelling under restricted conditions. This approach achieves a match between the resonance frequency and spatial position of the sample. A three-component hydrogel with salt ions was designed and synthesized to show the monitoring more clearly. The relationship between the hydrogel swelling and the frequency signal is revealed through the one-dimensional imaging. This method enables real-time monitoring and avoids changing the swelling environment of the hydrogel during contact. The accuracy of this method may reach the micron order. This finding provides an approach to the rapid and non-destructive detection of swelling, especially one-dimensional swelling, and may show the material exchange between the hydrogel and swelling medium.

## 1. Introduction

At present, nuclear magnetic resonance (NMR), including magnetic resonance imaging (MRI), has become one of the most important technologies in scientific research, and is extensively used in chemistry, biology, materials science and medical imaging [1]. NMR spectroscopy is widely used to identify the structure of organic natural products [2], to identify the chemical composition distribution and fracture mode of random and block copolymers [3], to screen drug targets corresponding to proteins [4], to predict protein torsion angle by chemical shift [5], to characterize the technology and water content in food processing [6], to measure the diffusion of oil in rubber [7], and to determine the internal microstructure of wood [8]. Although MRI is widely accepted as a powerful medical imaging modality, it has also been recently used for analytical measurements in materials systems [9,10]. Under MRI mode, the spin position is searched and radiofrequency (RF) pulses are scanned in the presence of a magnetic field gradient. When the spin signal is properly reunited through the gradient field, a time-domain signal is produced to reflect the spatial distribution of the spin of the whole sample at that amplitude. High-resolution spectral data and high-definition imaging can be provided, even in the presence of an obviously nonuniform magnetic field. In addition, spectral imaging can be measured without complex conditions by relying on multidimensional experiments. With the help of the auxiliary field gradient, the spin coordinates are converted into offsets in a one-to-one manner and mapped into frequencies. Although the resolution of the NMR peaks is sacrificed, the spatial positions are imprinted on the NMR line shapes [10].

A hydrogel is a hydrophilic polymer material with a three-dimensional polymer network, and chemically synthesized hydrogels are polymerized by monomers containing functional groups, such as hydroxyl and carbon double bonds [11]. The hydrophilicity, thermal stability, mechanical stability, biocompatibility, and responsiveness the material possesses are different in the swelling state. These properties make it useful for a number of applications in chemical engineering, drug delivery, tissue engineering, food and agriculture [12]. For example, silicone hydrogel is used in contact lenses. Polyacrylamide hydrogel is used as an absorbent for wound dressing [13]. PVA hydrogel is used in drug delivery applications and as a soft tissue replacement. [14]. The hydrogel swelling behavior in fluids is a special characteristic, and the diffusion of loose macromolecules in the swelling state plays an important role in these applications [15]. Polymer swelling includes the penetration of small molecules into the interior of larger polymer molecules, which leads to changes in polymer volume [16]. When the molecular weight and the degree of polymer crosslinking are higher, such as in chemicals with crosslinked hydrogels in contact with water or other solvents, the solvent cannot disperse further because the chemical bonds in the polymer chains have a binding effect on the small molecules of the solvent. Therefore, the polymer remains in the swelling process [17]. The degree and speed of swelling are important indices for investigation. For example, the swelling speed of resin absorbing water in diapers needs to be large enough to ensure there is no side leakage. However, the gasket of the mechanical seal rubber needs only a tiny degree of expansion or even no expansion to ensure dimensional stability.

The most common methods in the swelling test include the mass method, volume method, and length method. Taking the length method as an example, the swelling degree can be obtained by evaluating the ratio of the change in the height (or length) of a hydrogel sample before and after swelling:

Q = (h − h_0_)/h_0_ (1)

where Q is the degree of swelling, h_0_ is the height before swelling, and h is the height after swelling. Similarly, the principles of the mass method and volumetric method are similar to those of the length method (Supplementary S1). There are also some tests based on altered masses, such as centrifugation, filtration, tea bagging, and Prudential dextrin methods [18].

Although these traditional methods can easily test the degree of swelling, crosslinking density, and swelling curve of the polymer, they still have some disadvantages, such as low accuracy, cumbersome operation, and lack of continuous measurement. Usually, during the measuring process, the samples are always separated from the solvent for weighing or measurement. This process changes the gel’s environment and is very time-consuming. The low mechanical strength of some highly swellable hydrogels may result in their structural destruction during the weight measurement, which may result in significant errors. Thus, these methods are unable to precisely monitor the dynamic swelling process of specimens without contact [19]. In addition, the relationship between swelling rates and experimental temperature, time, and various other factors cannot be used to monitor these sensitive details [20].

To solve these problems, some improved methods have been put forward, such as the linear variable differential transformer (LVDT) [21], fluid-dynamic gauging [22] and nonintrusive inductance swelling instruments using a dynamic mechanical analyzer [23]. A customized sample cell and time-varying attenuated total reflection Fourier transform infrared spectroscopy were used to track the swelling processes in polymer films [24]. On the basis of the length method, researchers from the University of Cambridge used an opposed laser displacement sensor to measure the swelling, but the actual measurement range was limited by the length of the laser beam and severe warping [25]. The most recent method, proposed by Tang and colleagues, employed an aggregation-induced emission approach to suggest a new technique for the measurement of swelling properties in hydrogels [26]. The one-dimensional swelling of NMR has been less reported in examinations of the swelling of hydrogels. Lee reported that NMR imaging requires a three-dimensional gradient [27]. The proposed technique significantly enhanced the traditional method of swelling measurement during observations of the swelling process. However, the initial weight and size of the hydrogel should be carefully selected to avoid over-swelling. Due to the difficulties in managing the subtle changes in the hydrogel during the swelling process, it is not easy to investigate the influence of various other factors, such as temperature and time, on swelling.

To meet the demand for continuous noncontact measurements and detect the restricted swelling behavior, we present an NMR technique based on spin-space encoding and the gradient-broadening method (PROFILE), which can lead to the real-time detection of swelling behavior at the micron scale. By applying PROFILE, the relationship between proton density and signal intensity was obtained using one-dimensional imaging, and the height of the whole gel sample could be expanded in the frequency domain. Thus, the change in the height of the swelling gel corresponds to the change in frequency.

A three-component hydrogel containing barium chloride was designed and successfully synthesized to demonstrate the detection process. By using very dilute Na_2_SO_4_ solution as the solvent, the barium ions in the hydrogel react with Na_2_SO_4_, creating a precipitation layer on the interface between the gel and liquid. Because the precipitation layer contains far fewer hydrogen protons than the hydrogel and the solvent, the NMR signal intensity sharply decreases at the interface, forming a deep trough in the NMR spectra, which makes the interface position and the corresponding frequency in the spectra more obvious. With the swelling of the hydrogel, the interface moves and the frequency of the trough also moves. By continuously tracing the trough (the NMR spectra can be obtained by a single scan of up to 4 s), the position of the interface was obtained; therefore, the change in the length of the swelled hydrogel can be detected in real-time. The accuracy of this method could be on the micron order, which is much better than that of traditional methods. This method provides a new strategy for investigating the degree of swelling and swelling dynamics of hydrogels or other crosslinked polymer systems, especially tiny swelling systems. It may also have many potential applications in other correlating fields.

## 2. Result and Discussion

### 2.1. The Synthesis and Characterization of Hydrogels

In the absence of Ba^2+^, the PMAB gel can be synthesized by following the mechanism of radical polymerization, with PEGA, MEA, and AM as polymeric monomers in an aqueous solution and APS as initiators. The ^1^H-NMR spectra of the monomer and the synthesized gel are given in S2–S5. The monomer showed obvious double-bond peaks in the range of δ = 5.5 − 6.5, while the double bond disappeared in the polymer, and the state changed from liquid to solid, indicating that the gel was successfully synthesized. Although the three-component gel was successfully synthesized, the polymerization effect cannot be achieved in the presence of higher concentrations of ions, and the resulting gel is extremely uneven. There are two possible reasons for this phenomenon: one is ion inhibition and the other is the impact of APS. In the presence of higher concentrations of inorganic metal ions, the ions will have an effect on the initiator, which, in turn, will inhibit polymerization. To verify this, we attempted to reduce the ion concentration, but the lower ion concentration still could not form the gel. The second reason is that the persulfate in the APS at the early stage of the reaction initiates the reaction, producing a primary radical with a sulfate-like structure, which binds to Ba^2+^ and loses the initiation effect. Therefore, this reaction used azodiisobutyronitrile (AIBN) instead of APS as the initiator and used a smaller amount of the initiator at a lower temperature. This was carefully protected from light agitation, and gradient heating was used at later stages of gel formation to reduce bursting and bubble formation. The dosage ratio of the initiator and monomer, adjusted to the substance, was n(AIBN):n(MEA):n(PEGA):n(AM) = 1:133:100:270 (optimal ratio), which can be completely dissolved into a clear system before polymerization, and can be polymerized at a suitable rate to obtain a homogeneous gel without bubbles.

### 2.2. Gradient-Based Broadening Fingerprint Obtained by Line Shape Enhancement (PROFILE)

The resonance frequency ω_0_ of protons in the static magnetic field B_0_ can be expressed as:

ω_0_ = γB_0_ (2)

where γ is the ^1^H gyromagnetic ratio. The resonance frequency ω_0_ of the protons is constant and independent of the spatial position of the protons.

When the gradient field is applied in the direction of B_0_, the effective field B_eff_ of the protons can be written as:

B_eff_ = B_0_ + G_z_z (3)

where G_z_ is the amplitude of the gradient pulse; and z is the spatial position of the protons. Here, the effective field B_eff_ is not uniform and changes linearly with the space position z. The effective resonance frequency of protons ω_eff_ can be modified as:

ω_eff_ = γ(B_0_ + G_z_z) (4)

Therefore, the proton signal will be detected with a wide bandwidth while the gradient field is active and transformed in the magnitude mode; the NMR spectrum is a square-shaped spread. In the spectrum, the intensity of the spread signal is correlated with the spatial distribution of the proton density. The broadening range Δ*f* of the signal can be written as:

Δ*f* = γ × Gz × Δz (5)

**Figure 1:**
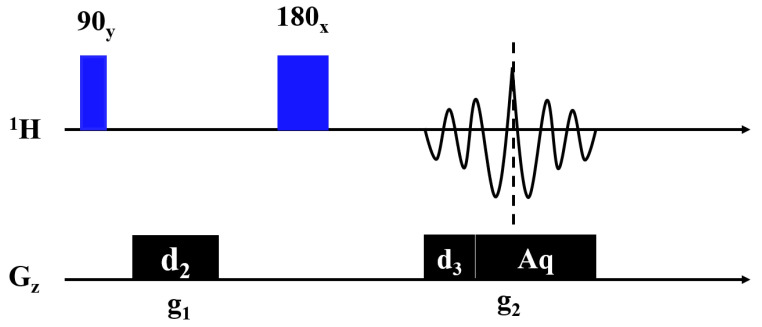
1D NMR spin-echo imaging sequence. The blue- and black-filled rectangles represent the hard pulses and gradient pulses, respectively. (90_y_: 90 degree hard pulse in y direction, 180_x_: 180 degree hard pulse in x direction, d_2_: the duration of gradient pulse g_1_, d_3_: the duration of gradient pulse g_2_, Aq: acquisition time )In order to collect a complete echo signal and avoid redundant noise, the durations can be set to: d_2_ = (d_3_ + aq)/2.

Figure 1 shows the 1D NMR spin-echo imaging sequence, which is used to measure the profile of an object. The radiofrequency (RF) pulses 90_y_ and 180_x_ were used to excite and refocus the NMR signals, respectively. The gradient pulse g_1_ was used to dephase the NMR signal, and the magnetic field gradient g_2_ was turned on for spatial encoding during acquisition. The profile of the object can be obtained by a Fourier transform of the temporal signal. The following sequence parameters were used in the studies: echo time (TE) = 8 ms; average number = 4; repetition time (TR) = 4 s; acquisition time = 6 ms; d_2_ = 4 ms; and d_3_ = 1 ms. A 10% maximum gradient strength $G_{max}$ was used in all experiments.

**Figure 2:**
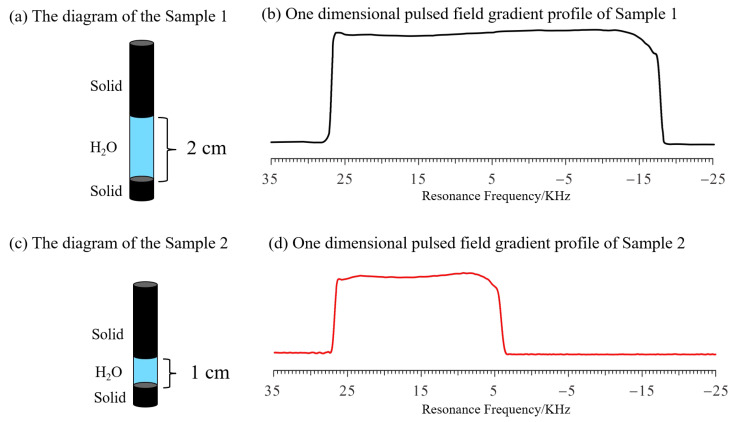
One−dimensional, pulsed−field gradient, nuclear magnetic resonance (1D NMR) profiles of deionized water in Shigemi tubes obtained by changing the sample height (Samples 1 and 2) while maintaining the same strength of the gradient pulses. (**a**) The diagram (Blue represents water and black represents a glass solid) of Sample 1 (220 μL deionized water; 2 cm height). (**b**) The gradient profile of sample 1 (10% maximum gradient strength $\;G_{max}$ (**c**) The diagram (Blue represents water and black represents a glass solid) of Sample 2 (110 μL deionized water; 1 cm height). (**d**) The gradient profile of sample 2 (10% maximum gradient strength). All profiles were obtained with 1 scan.

The Shigemi tube (see Figure 2a,c), constructed as two solid glass sections with no NMR signal at the top and bottom, was used to measure the maximum gradient field strength. Using deionized water as the standard sample, by applying PROFILE, the square-shaped spectrum was obtained and is shown in Figure 2b,d. For each experiment, the bottom interface was placed in the center of the probe, following Bruker’s NMR tube measuring cylinder. When water is added, the lower part of the tube is water and the upper part is solid (no proton). Thus, in the corresponding spectrum, the left part is water, and the right part is solid (no signal). The spectra of Sample 1 (220 μL; 2 cm high) are shown in Figure 2b; the frequency length $∆f_{1}$ of the profile at half the height of the gradient profile was about 45,780 Hz. According to Equation (5), we have

(6) $$∆f_{1}=\gamma \cdot G\cdot z_{1}=42.58\times 10^{6}\times 10\%\times G_{max}\times 2\times 10^{-2}$$

where z_1_ is the height of the water in Sample 1. The gradient that was applied is 10 percent of the maximum gradient.

From Equation (6), the maximum gradient strength $G_{max}$ could be calculated as 53.8 Gauss/cm.

Similarly, the spectra of Sample 2 (110 μL; 1 cm high) are shown in Figure 2d; the frequency length $f_{2}$ of the profile at half the height of the gradient profile was about 22,800 Hz, which is exactly half that of Δ*f*_1_. According to Equation (5), we also have

(7) $$∆f_{2}=\gamma \cdot G\cdot z_{2}=42.58\times 10^{6}\times 10\%\times G_{max}\times 1\times 10^{-2}$$

where z_2_ is the height of the water in Sample 2. The gradient that was applied is 10 percent of the maximum gradient.

From Equation (7), the $G_{max}$ could be calculated as 53.7 Gauss/cm, which is very similar to the result of Sample 1, and both results are in agreement with the data in the Bruker protocol. This result shows the reliability of this method.

### 2.3. Swelling Behavior of PMAB

**Figure 3:**
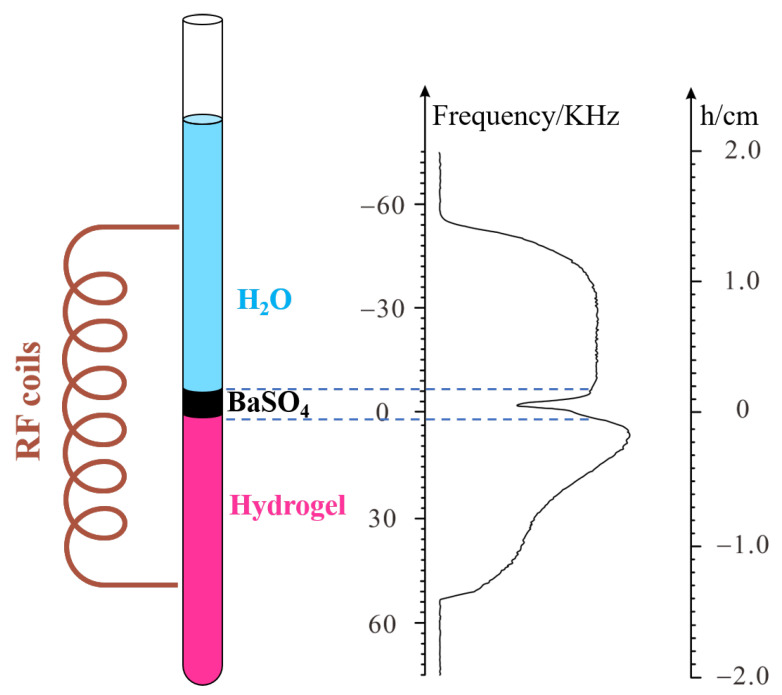
The spatial position of the NMR tube corresponds to the gradient profile. The brown represents the RF coil, and in the left NMR tube schematic diagram, the gel represents pink, the barium sulfate precipitate is black, and the water is blue from the bottom up.

After verifying the feasibility of the method, the swelling behavior of PMAB gel was studied using the PROFILE method. The interface was placed in the center of the probe, as described in the previous experiment. The schematic diagram is shown in Figure 3.

The NMR−spectra−based PROFILE can be obtained by a single scan (a scan takes up to 4 s) and is not sensitive to the field inhomogeneity. Each scan obtains a spectrum. Thus, the dynamic swelling behavior can be studied by continuous NMR scans or according to a designed time schedule. When there is only gel before adding the solvent, the spectra have a square-shaped profile (see Supplementary S7).

As mentioned above, the PMAB gel contains Ba^2+^, and diluted Na_2_SO_4_ solution was used as a swelling solvent. Both Ba^2+^ and Na^+^ are diamagnetic metal ions and have little effect on the NMR signal. The PMAB gel was directly synthesized in the NMR tube. When the Na_2_SO_4_ solution was added to the gel, barium sulfate was formed, and a precipitation layer was formed at the interface between the gel and solvent. Because barium sulfate has no hydrogen, the precipitation layer contains much less hydrogen than both the gel and solvent. When PROFILE pulse sequences are performed, the profile of the system can be reflected in the frequency domain. A deep trough appears at the position corresponding to the precipitation layer, which can serve as a sign to trace the swelling of the gel. As the swelling continues, the precipitation layer moves upwards, and the corresponding trough moves downwards.

**Figure 4:**
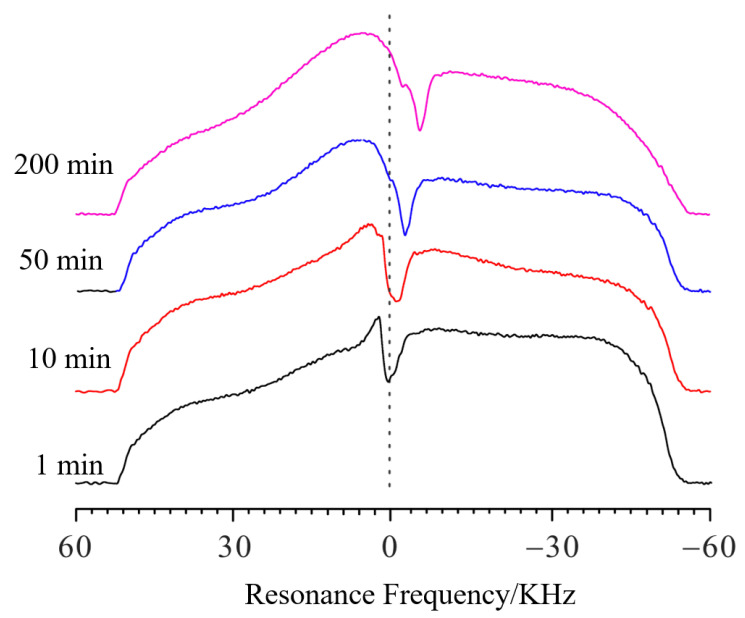
The NMR profile spectra of PMAB gels at different swelling times (25 °C). The black line represents 1 min, the red line represents 10 min, the blue line represents 50 min, and the pink line represents 200 min.

If there is only a gel before the addition of solvent, the spectral line shows an approximate rectangle. The slight distortion on the right side may be caused by the slight surface irregularities in the gel (see Supplementary S7). Figure 4 shows the spectra of swelling PMAB at different time points. The experimental parameters were the same as those in the verifying experiments. Compared with the spectra of pure gel, when the solvent was added, the profile of the spectra became a large square, with a deep trough at the interface. It is clear that as the swelling continued, the trough shifted toward the negative direction, which means that the PMAB gel swelled, and the interface moved upwards. In the verifying experiments, the relationship between the length change (Δ*h*) and the frequency change (Δ*f*) was calculated to be 4.36 μm/10 Hz.

Each profile spectrum on the left side is aligned and the frequency value is 52,400 Hz.

Then, the frequency value of the trough of the PMAB swelling spectrum after 1 min is 2759 Hz; the *f* values for 10 min, 50 min and 200 min of swelling are 1254 Hz, 167 Hz and −2174 Hz, respectively. If we set the frequency value of the trough of the PMAB swelling spectrum at 1 min as the standard, the Δ*f* values (compared to 1 min) for swelling periods of 10 min, 50 min and 200 min are −1505 Hz, −2592 Hz and −4933 Hz, respectively. Thus, the Δ*h* at different time points can be calculated as 656 μm, 1130 μm and 2151 μm, respectively, and the swelling at different time points can be obtained. The swelling degree of PMAB is quite low, making it difficult to detect with other traditional methods.

To obtain a higher resolution for the spectral characterization of hydrogen and investigate the material transfer, the PGAB gel was designed to be partially deuterated (to lock and shim the field); thus, the intensity of the spectra of the gel part is lower than that of the solvent part. When swelling began, the water diffused into the gel and made a ridge at the top of the gel. As the swelling continued, the ridge became broader, as the water diffused deeper into the gel. This shows that this method could be used to study substance exchange.

**Figure 5:**
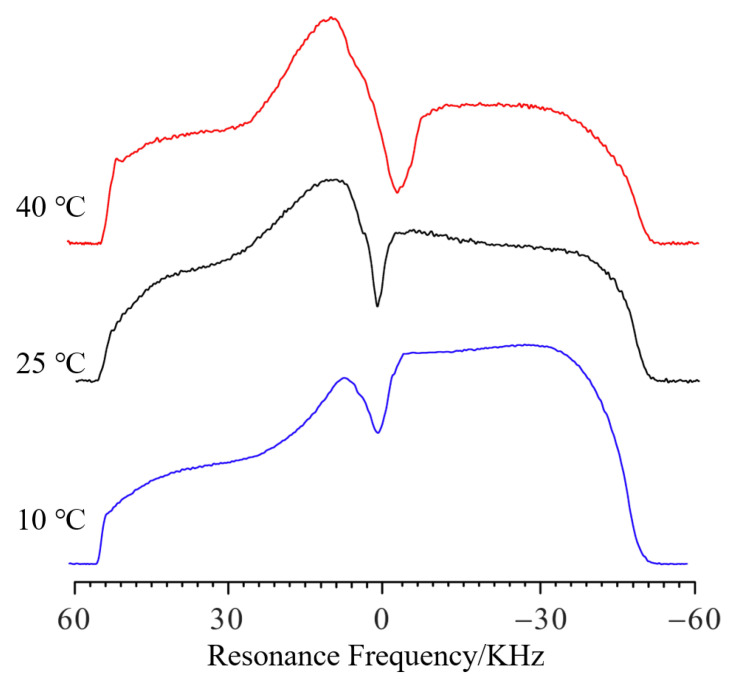
The NMR profile spectra of PMAB gels swelling at different temperatures but for the same amount of time (80 min). The blue line represents 10 °C, the black line represents 25 °C, and the red line represents 40 °C.

The influence of temperature on the swelling was also studied. Figure 5 shows the NMR profile spectra of PMAB gels swelling for 80 min at different temperatures. The frequency values *f* of that swelling for 10 °C, 25 °C and 40 °C were 1288 Hz, −1889 Hz and −5238 Hz, respectively. Compared to 10 °C, the Δ*f* values of that swelling for 25 °C and 40 °C were −3177 Hz and −6526 Hz, respectively. Thus, compared to 10 °C, the Δ*h* at different temperatures can be calculated as 1385 μm and 2845 μm, respectively, and the difference in swelling height at different temperatures can be obtained.

When the temperature was higher, the Δ*f* became higher for the same amount of swelling time, which means that the swelling speed is higher at higher temperatures. As the temperature rises, the gradual bulge on the left side of the curve indicates that the upper part of the water enters the gel faster, and the hydrogen signal further increases. A faster decline on the right side (height different from that of the baseline) also indicates that deuterium water from the gel enters the upper water layer.

**Figure 6:**
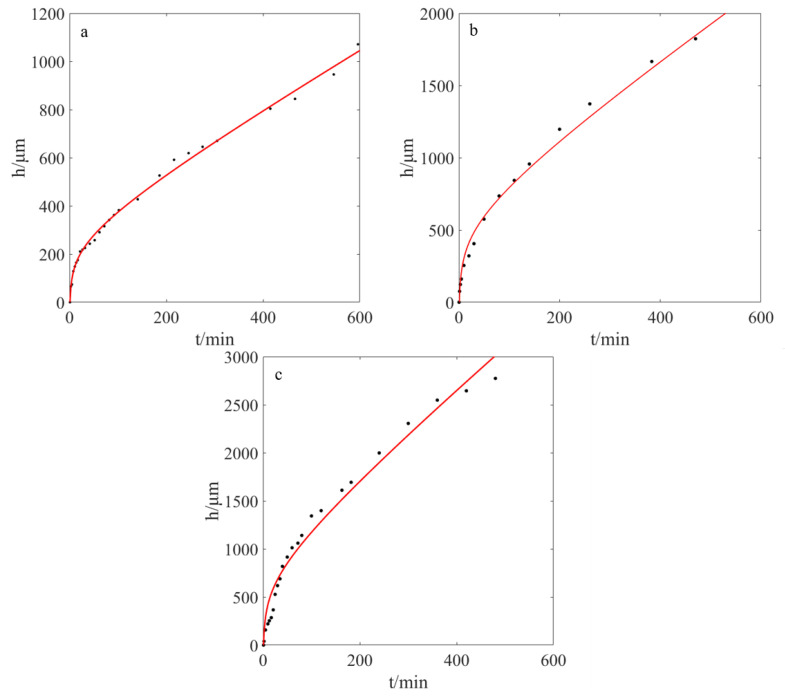
The relationship between gel height and swelling time at different temperatures. (**a**): 10 °C (**b**): 25 °C (**c**): 40 °C (the black dots are experiment data, and the red curves are simulated curves).

By transferring the Δ*f* into Δ*h*, the relationship between Δ*h* and swelling time can be observed, as shown in Figure 6, and the plot can be simulated by a logarithmic function:

Δ*h*(t) = a·log(t) + b·t (8)

where Δ*h* represents the height compared to the initial increase in the gel, *t* stands for time, and the units are microns and minutes, respectively. When the temperature is 25 °C, the resulting formula is Δ*h*(t) = 121.4·log(t) + 2.338·t and the fitting degree R is greater than 0.99. At other temperatures, the experimental data can also be simulated by Equation (8), as shown in Figure 6, and the simulated parameters are shown in Table 1.

The swelling process can be roughly divided into two phases: one segment is more of a logarithmic function and the remaining segment is more of a linear function. For example, at 40 °C, the portion that is closer to 200 min is closer to logarithmic expansion, and at 25 °C, the portion that is closer to 150 min is closer to logarithmic expansion. Finally, at 10 °C, the fraction that is closer to 100 min is closer to logarithmic expansion. The simulated function is similar to that previously reported by Luo [28].

## 3. Materials and Methods

### 3.1. Materials

2-Methoxyethyl acrylate (MEA, 98%), polyethylene glycol acrylate (PEGA, Mn = 480), and D_2_O (99.9% deuterated) were purchased from Sigma Aldrich, USA; acrylamide (AM, 99%) was purchased from Greagent; azodiisobutyronitrile (AIBN) (98%), and BaCl_2_·2H_2_O (99%) were purchased from Adamas; and ammonium persulfate (APS, 98%) was purchased from Sinopharm Chemical Reagent Co., Ltd. (Shanghai, China). Other reagents were of analytical grade purity. The above reagents were used directly without treatment.

### 3.2. Experiment

#### 3.2.1. The Synthesis of PEGA-MEA-AM Multi Component Gel

**Figure 7:**
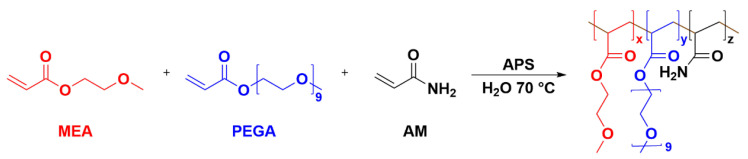
The synthesis of PEGA-MEA-AM multi-component gel. Red molecules represent the MEA fraction, blue molecules represent the PEGA fraction, and black molecules represent the AM fraction.

2-Methoxyethyl acrylate (1.20 g, 9.22 mmol) was added to the solvent of PEGA (3.0 g, 6.25 mmol), dissolved in water (10.40 g, 577.78 mmol) and mixed for 30 min to ensure that the compounds were completely dissolved. Acrylamide and a small amount of APS were then added to the solution and the mixture was stirred at 60 °C for 6 h. The hydrogel was cooled to room temperature. The synthetic pathway is shown in Figure 7.

#### 3.2.2. The Synthesis of PEGA-MEA-AM Gel with Salt

A small amount of 2-methoxyethyl acrylate (1.20 g, 9.22 mmol) was added to AIBN; the mixture was stirred at 15 °C for 30 min in the absence of light until the white solid was totally dissolved. PEGA (3.0 g, 6.25 mmol) was stirred in water (1.44 g, 80.00 mmol) for 30 min to completely dissolve the monomers. The mixture of MEA and AIBN was then added to the solution of PEGA. Next, BaCl_2_·2H_2_O (2.44 g, 10 mmol) was dissolved in water (7 g, 388.89 mmol), and AM (1.2 g, 16.88 mmol) was dissolved in water (2.0 g, 111.11 mmol). These were successively added to the mixture of MEA, AIBN and PEGA. The above solution was injected into the NMR tube and heated at 70 °C, then heated in a water bath by gradient heating for 8 h while remaining upright. The hydrogel was then allowed to cool to room temperature.

#### 3.2.3. The Synthesis of Deuterated Hydrogel

On the basis of the above reaction conditions and method, the deuterated hydrogel can be obtained by changing the water in the reaction into a mixture of deuterated water and water at a certain ratio. However, only some of the water was changed to a mixture of water (1.08 g, 60.00 mmol) and deuterated water (0.4 g, 60.00 mmol). The resulting hydrogel was named PMAB gel.

### 3.3. Characterization

One-dimensional ^1^H and ^13^C NMR spectra of the monomers and hydrogel samples were acquired with a Bruker 500 MHz AVANCE III NMR spectrometer. All the experiments were performed at 298 K and had no spin. ^1^H NMR experiments were acquired using the Bruker sequence “zg”. The acquisition parameters were as follows: time domain (number of data points), 39,998; dummy scans, 0; number of scans, 16; acquisition time, 1.99 s; delay time, 5 s; pulse width, 10 μs; spectral width, 19.99 ppm (10,000 Hz); FID resolution 0.500025 Hz; and digitization mode, digital. The total acquisition time was 1 min and 20 s. ^13^C NMR experiments were performed by optimizing a sequence that was modified to reduce the ringing effect and completely avoid ^1^H-^13^C coupling and NOE during relaxation. After measuring each carbon T_1_, a delay time (D_1_) equal to 5 × T_1_MAX (the longest relaxation time) was set to assure the complete relaxation of ^13^C nuclei. All these experimental conditions were used to make the carbon integral suitable for quantitative purposes. The experiments were acquired using the Bruker sequence “zgdc” (details are provided in the Supporting Information), and the acquisition parameters were consequently modified and set as follows: time domain (number of data points), 22,722; dummy scans, 0; number of scans, 64; acquisition time, 0.98 s; delay time, 10 s; spectral width, 301.16 ppm (37,878.789 Hz); FID resolution, 3.334 Hz; and digitization mode, digital.

## 4. Conclusions

In summary, a non-destructive testing method based on the NMR gradient-broadening profile was put forward. Using this method, gel swelling can be continuously observed at the micron level with high sensitivity and spatial resolution, and without requiring contact. By using PROFILE, the sample profile can be extended to the frequency domain. Under these experimental conditions, a 20 mm sample was extended to 45,780 Hz. Due to the high accuracy of NMR in the frequency domain (normally on the order of Hz), the hydrogel detection accuracy of this method should be at the micron scale, which is much higher than that of most other methods. A three-component hydrogel containing salt ions was synthesized. By choosing the proper swelling medium, the produced precipitate can be used to show the position of the interface between gel and liquid. By performing continuous NMR scans, the swelling behavior can be studied in real time. Thus, this method has obvious advantages when small swelling occurs and in real-time studies. Although the PROFILE was confirmed to be a powerful technique, it still has some restrictions. The RF coil length of the NMR instrument is limited and the maximal swelling height should not exceed that of the coil; thus, the initial sample length should be properly selected.

As an additional advantage, the diffusion of water into the gel was observed, which provides further possibilities to use this method in gel studies, such as in studies of the material exchange between the gel and solvent, dynamics of the solvent molecules, etc. A related investigation is ongoing.

## Figures and Tables

**Table 1:** Influencing factors during swelling and fitting (with 95% confidence bounds) at different temperatures.

	Fitting Formula	Δ*h*(t) = a·log(t) + b·t		
T/°C		a	b	R
10		56.33 (±3.03)	1.142 (±0.058)	0.9961
25		121.4 (±15.1)	2.338 (±0.273)	0.9920
40		164.3 (±25.5)	4.159 (±0.585)	0.9753

## Data Availability

The original data presented in this study are available from the authors.

## Supplementary Materials

The following supporting information can be downloaded at: https://www.mdpi.com/article/10.3390/molecules28073116/s1, Supplementary S1, The principle of the volumetric method. Supplementary S2, The ^1^H NMR spectrum of PEGA: Figure S1. The ^1^H NMR spectrum of PEGA. Supplementary S3, The ^1^H NMR spectrum of AM: Figure S2. The ^1^H NMR spectrum of AM. Supplementary S4, The ^1^H NMR spectrum of MEA: Figure S3. The ^1^H NMR spectrum of MEA. Supplementary S5, The ^1^H NMR spectrum of PMAB gel: Figure S4. The 1H NMR spectrum of PMAB gel. Supplementary S6, The ^13^C and DEPT-135^o^ NMR spectrum of gel: Figure S5. The ^13^C and DEPT-135^o^ NMR spectra of PMAB gel. Supplementary S7, The NMR profile sperctra of pure PMAB gel without solvent: Figure S6. The NMR profile spectra of pure PMAB gel without solvent.

## Sample Availability

Samples of the compounds are not available from the authors.
