# Supplementary material for: Spin-Space-Encoding Magnetic Resonance Imaging: A New Application for Rapid and Sensitive Monitoring of Dynamic Swelling of Confined Hydrogels
Source: Molecules. 2023 Mar 30;28(7):3116. doi: 10.3390/molecules28073116 (PMC10096351; doi:10.3390/molecules28073116)
Supplement: Supplementary file 1 [file molecules-28-03116-s001.zip › molecules-2300076-supplementary.pdf]

molecules MDPI

Support Information

Supplementary S1

The principle of the volumetric method

The current mainstream ways of measuring swelling include the mass method, which evaluates the ratio of the change in mass of a formula sample by the ratio of the change in mass before and after swelling. In the mass of the specimen, m, to the original mass of the specimen before it was swollen is the degree of swelling of the polymer measured. The formula for the degree of swelling is $\boldsymbol{Q} = (\mathbf{m} - \boldsymbol{m0})/\boldsymbol{m0}$ In this formula, Q is the degree of swelling, m is the mass after swelling, and m_0 is the mass of the cross-linked polymer before swelling. However, in the swelling process, the surface of the material must be dried and separated from the solvent before weighing. In addition, there are a series of methods for measuring the swelling of cross-linked polymers based on altered masses such as centrifugation, filtration, tea bagging and Prudential dextrin methods.

The formula for the degree of swelling is $\boldsymbol{Q} = \boldsymbol{\rho}\Delta\mathbf{V}/\boldsymbol{m0}$. In this formula, ΔV is the change in volume of the cross-linked polymer specimen, ρ is the solvent density and m_0 is the mass of the cross-linked polymer is the cross-linked polymer specimen before swelling. Alternatively, the swelling is expressed directly as a ratio of volumes, making the formula for the degree of swelling become. $\boldsymbol{Q} = \Delta\mathbf{V}/\boldsymbol{V0}$ In this formula, ΔV is the change in volume of the cross-linked polymer specimen, and V_0 is the volume of the cross-linked polymer specimen before swelling. The volumetric method can obtain the results by measuring the volume change of the solvent or the cross-linked polymer specimen. Thus, the degree of swelling can be obtained by separating the swollen cross-linked polymer from the solvent and measuring the volume change of the solvent, or by measuring the volume change of the cross-linked polymer specimen using a swelling meter.

Supplementary S2

The ^{1}H NMR spectrum of PEGA

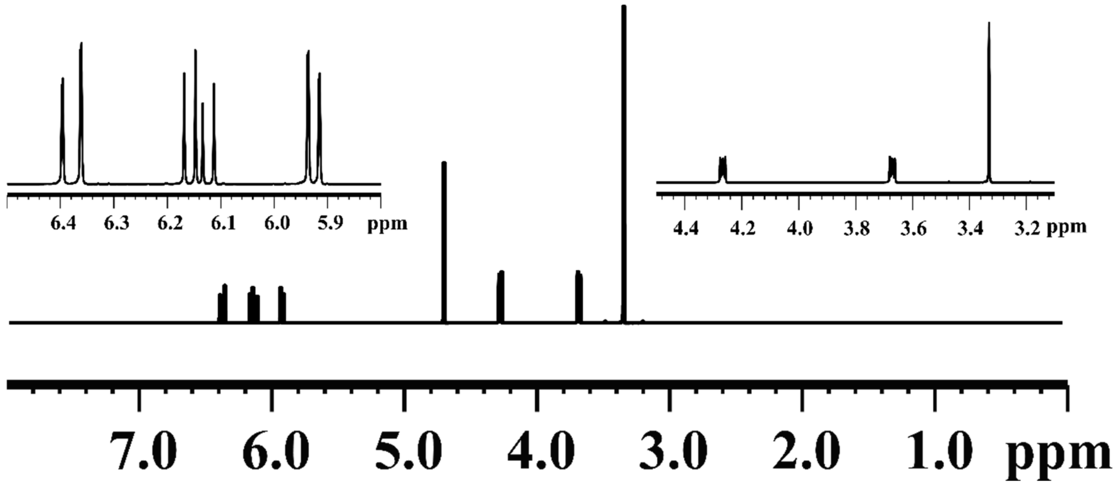

Figure S1: The ^{1}H NMR spectrum of PEGA

Supplementary S3

The ^{1}H NMR spectrum of AM

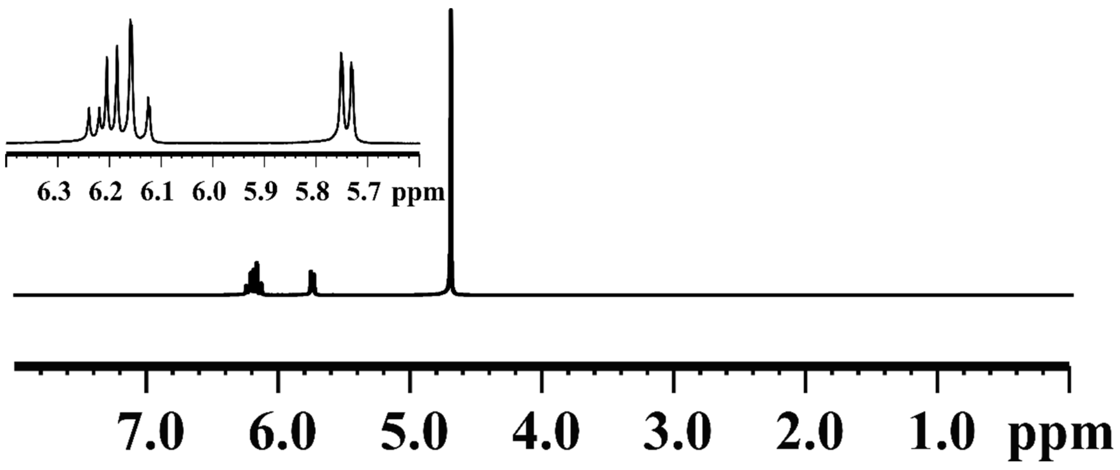

Figure S2: The ^{1}H NMR spectrum of AM

Supplementary S4

The ^{1}H NMR spectrum of MEA

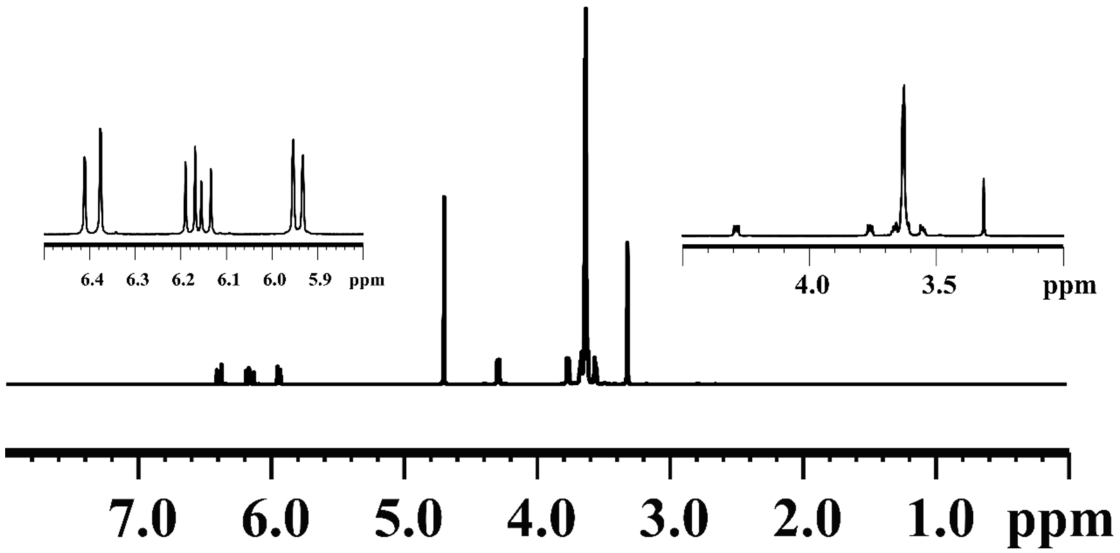

Figure S3: The ^{1}H NMR spectrum of MEA

Supplementary S5

The ¹H NMR spectrum of PMAB gel

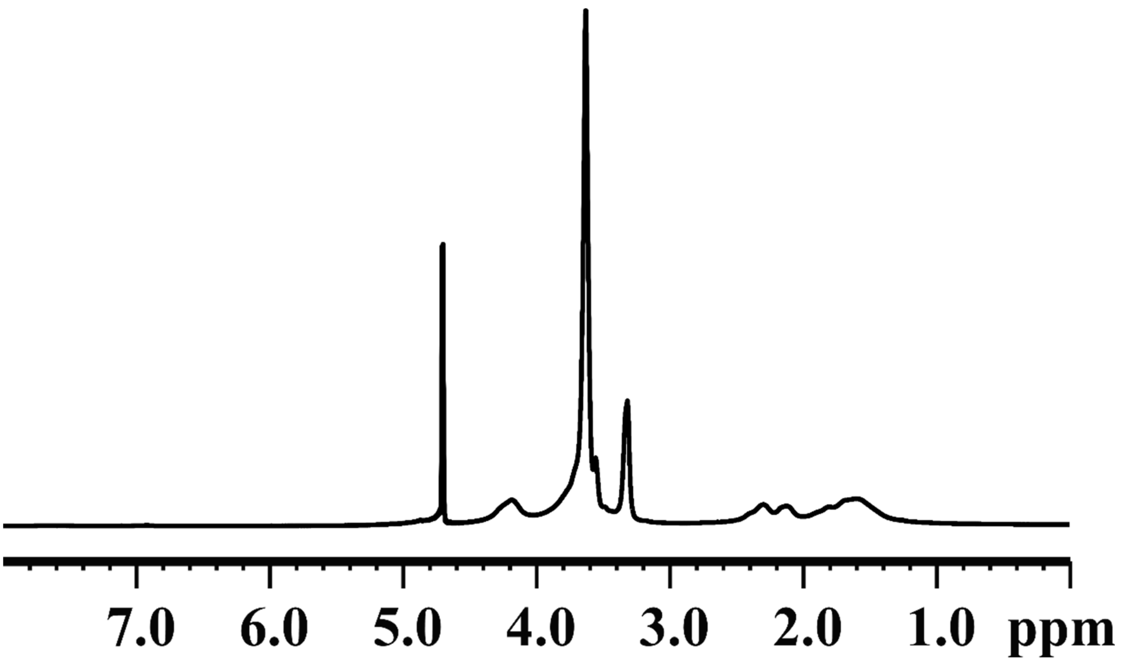

Figure S4: The ¹H NMR spectrum of PMAB gel

Supplementary S6

The ¹³C and DEPT-135° NMR spectrum of gel

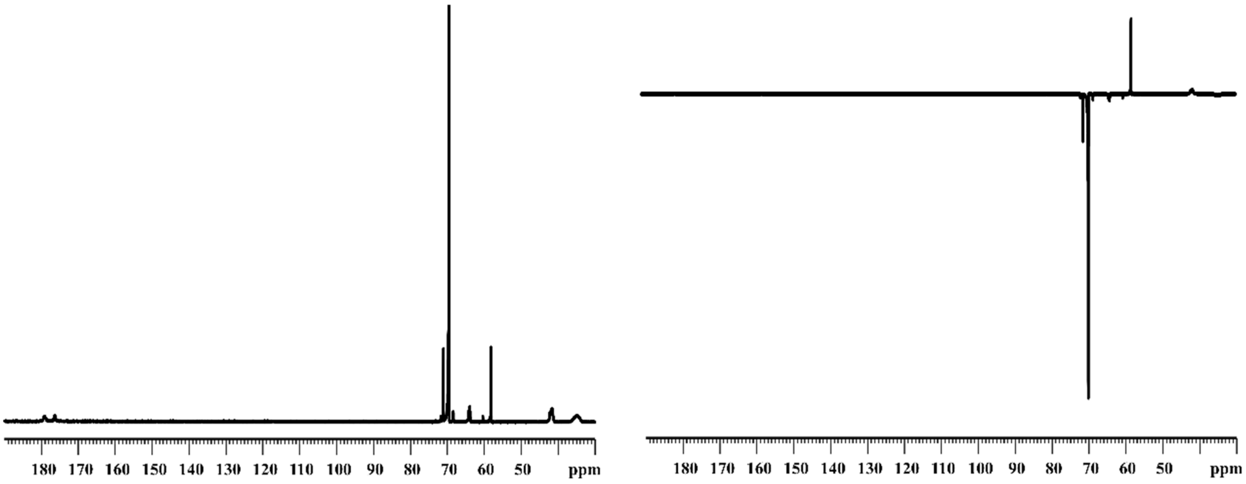

Figure S5: The ¹³C and DEPT-135° NMR spectra of PMAB gel

Supplementary S7

The NMR profile sperctra of pure PMAB gel without solvent

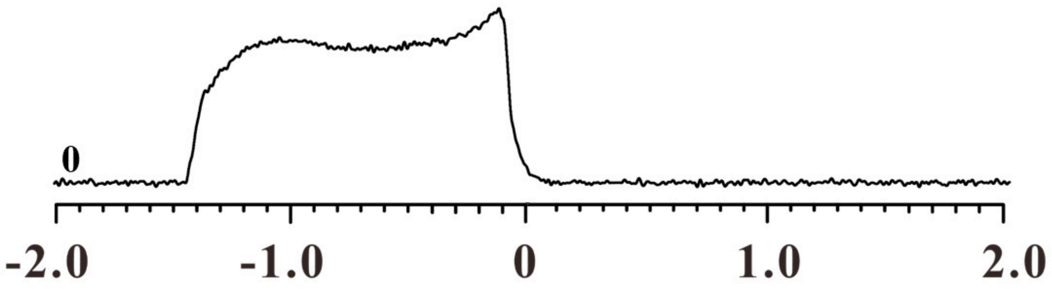

Figure S6: The NMR profile spectra of pure PMAB gel without solvent
